# Availability and accuracy of occupation in cancer registry data among Florida firefighters

**DOI:** 10.1371/journal.pone.0215867

**Published:** 2019-04-30

**Authors:** Laura A. McClure, Tulay Koru-Sengul, Monique N. Hernandez, Jill A. Mackinnon, Natasha Schaefer Solle, Alberto J. Caban-Martinez, David J. Lee, Erin Kobetz

**Affiliations:** 1 Sylvester Comprehensive Cancer Center, University of Miami Miller School of Medicine, Miami, FL, United States of America; 2 Department of Public Health Sciences, University of Miami Miller School of Medicine, Miami, FL, United States of America; 3 Florida Cancer Data System, Sylvester Comprehensive Cancer Center, University of Miami Miller School of Medicine, Miami, FL, United States of America; 4 Department of Medicine, University of Miami Miller School of Medicine, Miami, FL, United States of America; University of Oxford, UNITED KINGDOM

## Abstract

**Objectives:**

Occupational exposures significantly contribute to the risk of adverse cancer outcomes, and firefighters face many carcinogenic exposures. Occupational research using cancer registry data, however, is limited by missing and inaccurate occupation-related fields. The objective of this study is to determine the frequency and predictors of missing and inaccurate occupation data for a cohort of career firefighters in a state cancer registry.

**Methods:**

We conducted a linkage between data from the Florida Cancer Data System (1981–2014) and the Florida State Fire Marshal’s Office (1972–2012). The percentage and the odds of having a firefighting-related occupation code in the cancer record were calculated, adjusting for other occupation and cancer-related factors.

**Results:**

Among 3,928 career firefighters, nearly half (47%) were missing a registry-dervived occupation code and only 17% had a firefighting-related code. Males were more likely to have a firefighting-related code (OR = 2.31;95%CI: 1.41–3.76), as were those with more recent diagnoses (OR_1992-2002_ = 2.98;95%CI: 1.57–5.67; OR_2003-2014_ = 11.40;95%CI: 6.17–21.03), and those of younger ages (OR_45-64y_ = 1.26;95%CI: 1.03–1.54; OR_20-44y_ = 2.26;95%CI: 1.73–2.95).

**Conclusions:**

Accurate occupation data is key for identifying increased risk of advserse cancer outcomes. Cancer registry occupation fields, however, are overwhelmingly missing for firefighters and are missing disproportionally by sociodemographic and diagnosis characteristics. This study highlights the lack of accurate occupation data available for hypothesis-driven cancer research. Cancer registry linkage with external occupational data sources represents an essential resource for conducting studies among at-risk populations such as firefighters.

## Introduction

Occupational exposures play an important role in the risk of cancer and cancer death[1]. Indeed, it is estimated that occupational exposures contribute to 40,000 new cancer cases and 20,000 cancer deaths each year in the United States (US)[2]. A recent review suggests that 2–8% of all cancers may be attributable to occupational exposures[3]. Firefighters, for example, face many hazardous and potentially carcinogenic exposures in their jobs[1, 4], and they have been shown to be at increased risk of many cancers[5–7]. Measuring the impact of occupational exposures, however, can be a challenge, particularly given the latency of cancer development[3].

Cancer registry data are vital for the surveillance of cancer trends, but the occupation fields are often incomplete and inaccurate[8, 9]. Via medical record abstraction, participants of the National Program of Cancer Registries (NPCR) collect text indicating usual occupation (“type of job patient engaged in for the greatest number of working years”) and text indicating usual industry (“type of business or industry where patient worked in his or her usual occupation”)[5]. These data are frequently retrieved from narrative fields in the medical record, which take no standard form, and coding often requires extensive manual review and processing[10]. Not only are these data frequently missing, they are often missing in a differential fashion based on facility, provider, insurance status, and occupation[4].

The objective of the current study is to determine the frequency and predictors of missing and inaccurate occupation data for a cohort of career firefighters in a state cancer registry.

## Materials and methods

The Florida Cancer Data System (FCDS) is the legislatively mandated, population-based cancer registry for the state of Florida in operation under the Florida Department of Health as part of NPCR. It has collected all newly diagnosed cancer cases in Florida since 1981. The Florida State Fire Marshal’s Office has collected firefighter certification records for all firefighters in the state since 1972 when certification was mandated.

Using fastLink[11], FCDS performed a probabilistic linkage between 1981–2014 FCDS data and 1972–2012 data from the Florida State Fire Marshal. The resulting enriched linked dataset contained 3,928 cases with at least one incident cancer. Using this dataset we determined the number of cancer cases that had any 2010 Census occupation code in their FCDS registry record and the number that had a firefighting-related code. The firefighting-related occupations included the following five codes: firefighters 3740, first-line supervisors of firefighting and prevention workers 3720, fire inspectors 3750, and emergency medical technicians and paramedics 3400. The last code (3400) was included because many Florida firefighters are additionally certified in this area. We included in our sample, only firefighters considered to be “career” firefighters, rather than volunteers, as defined by guidance from the State Fire Marshal’s Office.

We conducted descriptive data analyses to determine the number and percent of linked firefighter cancer cases that had any occupation code or any of the four firefighting-related occupation codes listed in their first primary malignant cancer registry record. These results were evaluated by demographic and cancer diagnosis characteristics (gender, race/ethnicity, and year and age of cancer diagnosis). The same descriptive analysis was performed on the FCDS database for first primary malignant cancers diagnosed between 1981 and 2014. We also conducted univariate and multivariable logistic regression analyses with the presence of a cancer registry-derived firefighting-related occupation code (yes/no) as the dependent variable. The multivariable regression model was adjusted for gender (male/female), ethnicity (Hispanic, non-Hispanic, unknown), race (White, non-White, unknown), age at cancer diagnosis (20-44y, 45-64y, 65y+), and year of cancer diagnosis (1981–1991, 1992–2002, 2003–2014). Unadjusted odd ratios (OR) and adjusted odds ratios (aOR) and corresponding 95% confidence intervals (95%CI) were calculated. Data management and statistical analysis were completed using SAS Enterprise Guide software, Version 5.1(SAS Institute Inc. Cary, NC, USA). This study was approved by the Institutional Review Boards of the Florida Department of Health and the University of Miami.

## Results

Of the 3,928 firefighters with cancer in our enriched linked dataset, almost half were missing an occupation code of any kind (n = 1,848, 47.0%) in the cancer registry record for their first primary cancer (Table 1). Only 679 (17.3%) had a firefighting-related occupation code; of these, 79.2% (n = 538) were listed as firefighters, 9.9% (n = 67) were emergency medical technicians and paramedics, 7.8% (n = 53) were first-line supervisors of firefighting and prevention workers, and 3.1% (n = 21) were fire inspectors. The remainder of linked cases (n = 1,401, 35.7%) had an occupation code other than firefighting, which included 1,071 (27.3% of total sample) listed as “unemployed” or “retired” and 330 (8.4%) with any other occupation.

**Table 1:** Presence of any occupation code and firefighting-related occupation code among linked career firefighter cancer cases and the Florida Cancer Data System (FCDS) overall, first primary malignant cancers only, 1981–2014.

	Linked Firefighters n = 3,928		FCDS n = 2,622,219	
	n	%	N	%
**Missing Occupation Code**	1,848	47.0	1,837,467	70.1
**Any Occupation Code**	2,080	53.0	784,752	29.9
** Non-Firefighting Code**	1,401	35.7	782,932	99.9
** Any Firefighting-Related Code**	679	17.3	1,775	0.1
** 3740**: Firefighters	538	79.2	1,341	73.7
** 3720**: First-line supervisors of firefighting and prevention workers	53	7.8	152	8.3
** 3750**: Fire inspectors	21	3.1	47	2.6
** 3400**: Emergency medical technicians and paramedics	67	9.9	235	12.9

Compared to our linked firefighters, a greater percentage (70.1%, n = 1,837,467) of the FCDS dataset from 1981–2014 was missing an occupation code of any kind among first primary cancers (Table 1). There were a total of 1,775 cases with a firefighting-related occupation code in FCDS for this time period, the difference between this and our linked number (n = 679) likely being accounted for by firefighters that were certified in states other than Florida and thus were not linked to the Florida State Fire Marshal data.

In the linked dataset, a firefighting-related code was found in greater proportion among Hispanics/unknown ethnicity (22.4% vs. 17.0% among non-Hispanics). The percentage with a firefighting-related code decreased with age at diagnosis (20-44y: 22.4%; 45-64y: 16.9%; 65y+: 15.2%) and increased with year of diagnosis (1981–1991: 3.1%, 1992–2002: 7.7%; 2003–2014: 23.4%) (Table 2).

**Table 2:** Sociodemographic characteristics by presence of a firefighting-related occupation code among linked career firefighter cancer cases, 1981–2014.

	All		Firefighting-related code					
			Yes			No		
	n	col%	n	row%	col%	n	row%	col%
**Total**	3,928	100	679	17.3	100	3,249	82.7	100
**Gender**								
Male	3,760	95.7	659	17.5	97.1	3,101	82.5	95.4
Female	168	4.3	20	11.9	2.9	148	88.1	4.6
**Ethnicity**								
Non-Hispanic	3,736	95.1	636	17.0	93.7	3,10	83.0	95.4
Hispanic*	192	4.9	43	22.4	6.3	149	77.6	4.6
**Race**								
White	3,677	93.6	628	17.1	92.5	3,049	82.9	93.8
All Others**	251	6.4	51	20.3	7.5	200	79.7	6.2
**Year of Cancer DX**								
1981–1991	350	8.9	11	3.1	1.6	339	96.9	10.4
1992–2002	1,086	27.6	84	7.7	12.4	1,002	92.3	30.8
2003–2014	2,492	63.4	584	23.4	86.0	1,908	76.6	58.7
**Age groups at Cancer DX**								
20-44y	616	15.7	138	22.4	20.3	478	77.6	14.7
45-64y	2,155	54.9	365	16.9	53.8	1,790	83.1	55.1
65y+	1,157	29.5	176	15.2	25.9	981	84.8	30.2
**Age at Cancer DX (years)**								
mean (SD)	57.2 (12.6)		55.6 (13.3)			57.6 (12.4)		
median (25^th^%, 75^th^%)	58.0 (49.0, 66.0)		56.0 (47.0, 65.0)			58.0 (50.0, 66.0)		
min-max	20.0–94.0		22.0–89.0			20.0–94.0		

Col%: column percentages; SD: standard deviation; min: minimum; max: maximum; DX: diagnosis

*This category includes 35 cases with unknown ethnicity

**This category includes 26 cases with unknown race

Adjusted logistic regression modeling results (Table 3) show a significantly increased likelihood of having a firefighting-related occupation code among males (vs. females, aOR = 2.31, 95%CI: 1.41–3.76), those diagnosed in later years (vs. 1981–1991; aOR_1992-2002_ = 2.98, 95%CI: 1.57–5.67; aOR_2003-2014_ = 11.40, 95%CI: 6.17–21.03), and those diagnosed at younger ages (vs. 65+y; aOR_45-64y_ = 1.26, 95%CI: 1.03–1.54; aOR_20-44y_ = 2.26, 95%CI: 1.73–2.95).

**Table 3:** Univariate and multivariable logistic regression models for odds of having a firefighting-related occupation code in the linked career firefighter cancer cases, 1981–2014.

Variables	Univariate		Multivariable	
	OR (95%CI)	p-value	aOR (95%CI)	p-value
**Gender**				
** **Female	1 (ref)		1 (ref)	
** **Male	1.57 (0.98–2.53)	0.06	2.31 (1.41–3.76)	<0.001
**Ethnicity**				
** **Non-Hispanic	1 (ref)		1 (ref)	
** **Hispanic	1.67 (1.15–2.41)	<0.01	1.36 (0.93–2.01)	0.12
** **Unknown	0.46 (0.14–1.50)	0.20	0.40 (0.11–1.42)	0.16
**Race**				
** **White	1 (ref)		1 (ref)	
** **Non-White	1.25 (0.89–1.75)	0.20	1.04 (0.74–1.47)	0.83
** **Unknown	1.16 (0.43–3.08)	0.77	1.22 (0.42–3.54)	0.71
**Year of Cancer DX**				
** **1981–1991	1 (ref)		1 (ref)	
** **1992–2002	2.58 (1.36–4.90)	<0.01	2.98 (1.57–5.67)	<0.001
** **2003–2014	9.43 (5.14–17.31)	<0.001	11.40 (6.17–21.03)	<0.001
**Age groups at Cancer DX**				
** **65y+	1 (ref)		1 (ref)	
** **45-64y	1.14 (0.93–1.38)	0.20	1.26 (1.03–1.54)	0.03
** **20-44y	1.61 (1.26–2.06)	<0.001	2.26 (1.73–2.95)	<0.001

DX: diagnosis; OR: crude odds ratio; aOR: adjusted odds ratio; 95%CI: 95% confidence interval; the multivariable logistic model included all the variables listed in the table.

## Discussion

This study confirmed the overwhelming lack of accurate data in the occupation field of cancer registry records. While the linked firefighter cancer cases had a greater proportion of any occupation code present than in the FCDS dataset overall (53% vs. 30%), at only half of the study population, this represent a very poor source of data for occupational research. The greater proportion of codes present for our linked cases may be an example of reporting bias among occupations for which there is a known risk of cancer, as has been shown previously[4].

In addition to missing data, 8.4% of our firefighters had an occupation other than firefighting or unemployed in their cancer record. This may be because their longest held job was something other than a firefighting occupation, or it may be that the code is simply not an accurate representation of their occupation. The high percentage of those listed as unemployed or retired (27.3%) in our sample is likely due to the fact that data in the cancer registry record are obtained at the time of diagnosis, and given that a greater proportion of cancers are diagnosed in older age, cases are often unemployed or retired at that point[10]. Occupational fields are supposed to represent the longest held position, but it is clear that this is not always the case. Given that those of older age are both more likely to be out of the workforce and be diagnosed with cancer, the category of “unemployed”, therefore represents a greater source of inaccuracy in the occupation field than, say, mislabeling a firefighter as an engineer.

Our study also confirms that the presence of occupation codes varies by sociodemographic characteristics. Given the comparatively small number of female firefighters, it makes sense that they may be less likely to have this reported in their cancer record. It also makes sense that cases diagnosed in recent years would have more accurate occupation information as cancer registry data collection practices have improved over time. Similarly, those diagnosed at younger ages are also more likely to still be working and so have an accurate occupation reported.

The main concern with occupational data in the cancer registry is that the data abstracted is subject to the information available in the medical record, and the text in these fields are not recorded in any standardized or routine manner. Completeness and accuracy of occupation data in cancer registries and medical records is not likely to see dramatic improvements given the time and resource constraints placed on registries and healthcare facilities. The growing implementation of electronic health records has the potential to somewhat improve the ability to collect accurate occupation data, but there will be a need for the development of standardized fields in these electronic heath systems for any improvement to be realized.

A recent study of eight NPCR states employed the web-based NIOSH Industry and Occupation Computerized Coding System (NIOCCS), developed by the National Institute for Occupational Safety and Health[12, 13], to translate text into standardized industry and occupation codes[10]. Results indicated that this was a useful tool for improving occupation fields; however, registries reported the need for increased training to collect higher quality information to be either coded by registrars or auto-coded by the web-based tool. These types of tools are valuable resources, particularly when looking at all occupations combined, but they are limited in their usefulness when occupation information is simply missing from the medical record. In this study, for example, 50% of cases were missing occupation codes, and 24% were missing any text on occupation. Similarly, in a California study evaluating cancer among firefighters, 50% of registry records were missing data on industry and occupation[4, 14].

Data linkages such as the one conducted in our study provide a valuable and efficient alternative to improving completeness without the need for collecting further data or additional cancer registrar training. Additionally, because occupation data is often missing differentially[14], linkage may offer a less biased sample from which to conduct research. Data linkage has the further advantage of providing new fields with which to conduct research. This is true for our current linkage with firefighter data as well as a previous linkage conducted by our study team between FCDS and the National Health Interview Survey, which allowed for the examination of health behaviors, risk factors, and cancer screening among cancer cases[15, 16].

This study should be evaluated with the following limitation in mind: our sample represents Florida firefighters only, and while occupation-related data is overwhelmingly missing in cancer registries in general, it may be missing disproportionally by different characteristics in other registries than those seen here. A strength of our study is the ability to capture every cancer among Florida firefighters going back to 1981, which provides us with a large sample size with which to conduct hypothesis-driven research. A second strength was that we were able to include only career firefighters in the study sample. This allowed us to remove any bias that may have been introduced by including volunteers, who would have likely had a different main occupation code, when listed. It is important to note, however, that volunteers still face hazardous exposures and potentially increased risk of cancer [17–19].

In conclusion, occupational research using cancer registry data is limited by inaccurate and missing fields on occupation. Not only are these fields missing, they are missing disproportionally by case characteristics such as gender, age at diagnosis, and year of diagnosis. Data linkage is a strategy of particular benefit for specific populations with known exposures, such as firefighters. Linkage of firefighter data and cancer registry data on a national level would offer unparalleled advantage in providing accurate occupational information for this at-risk population.

## References

[1] World Health Organization International Agency for Research on Cancer. IARC Monographs on the Evaluation of Carcinogenic Risks to Humans, Volume 98: Painting, Firefighting, and Shiftwork Lyon, France2010 [Available from: https://monographs.iarc.fr/ENG/Monographs/vol98/mono98.pdf PMC4781497 21381544

[2] National Institute for Occupational Safety and Health (NIOSH). Cancer, Reproductive, Cardiovascular, and Other Chronic Disease Prevention Program Updated December 6, 2017 [Available from: https://www.cdc.gov/niosh/programs/crcd/description.html.

[3] Purdue MP, Hutchings SJ, Rushton L, Silverman DT. The proportion of cancer attributable to occupational exposures. Ann Epidemiol. 2015;25(3): 188–92. 10.1016/j.annepidem.2014.11.009 25487971 PMC4631263

[4] Silver SR, Tsai RJ, Morris CR, Boiano JM, Ju J, Scocozza MS, et al Codability of industry and occupation information from cancer registry records: Differences by patient demographics, casefinding source, payor, and cancer type. Am J Ind Med. 2018;61(6): 524–32. 10.1002/ajim.22840 29574892

[5] Department of Health and Human Services Centers for Disease Control and Prevention National Institute for Occupational Safety and Health. A Cancer Registrar's Guide to Collecting Industry and Occupation: DHHS (NIOSH) Publication No. 2011–173; 2011 [Available from: https://www.cdc.gov/niosh/docs/2011-173/pdfs/2011-173.pdf.

[6] LeMasters GK, Genaidy AM, Succop P, Deddens J, Sobeih T, Barriera-Viruet H, et al Cancer risk among firefighters: a review and meta-analysis of 32 studies. J Occup Environ Med. 2006;48(11): 1189–202. 10.1097/01.jom.0000246229.68697.90 17099456

[7] Ma F, Fleming LE, Lee DJ, Trapido E, Gerace TA. Cancer incidence in Florida professional firefighters, 1981 to 1999. J Occup Environ Med. 2006;48(9): 883–8. 10.1097/01.jom.0000235862.12518.04 16966954

[8] Levy J, Brooks D, Davis L. Availability and quality of industry and occupation information in the Massachusetts Cancer Registry. Am J Ind Med. 2001;40(1): 98–106. 11439402 10.1002/ajim.1076

[9] Weiss NS, Cooper SP, Socias C, Weiss RA, Chen VW. Coding of Central Cancer Registry Industry and Occupation Information: The Texas and Louisiana Experiences. J Registry Manag. 2015;42(3): 103–10. 27028094

[10] Freeman MB, Pollack LA, Rees JR, Johnson CJ, Rycroft RK, Rousseau DL, et al Capture and coding of industry and occupation measures: Findings from eight National Program of Cancer Registries states. Am J Ind Med. 2017;60(8): 689–95. 10.1002/ajim.22739 28692191 PMC5769461

[11] Enamorado T, Fifield B, Imai K. Using a Probabilistic Model to Assist Merging of Large-Scale Administrative Records. American Political Science Review. 2017.

[12] Centers for Disease Control and Prevention National Institute of Occupational Safety and Health (NIOSH). The NIOSH Industry and Occupation Computerized Coding System (NIOCCS) 2018 [Available from: https://wwwn.cdc.gov/nioccs3/.

[13] National Institute for Occupational Safety and Health. Simplify: How to quickly code industry and occupation data 2018 [Available from: https://blogs.cdc.gov/niosh-science-blog/2018/10/04/nioccs/.

[14] Tsai RJ, Luckhaupt SE, Schumacher P, Cress RD, Deapen DM, Calvert GM. Risk of cancer among firefighters in California, 1988–2007. Am J Ind Med. 2015;58(7): 715–29. 10.1002/ajim.22466 25943908 PMC4527530

[15] McClure LA, Miller EA, Tannenbaum SL, Hernandez MN, MacKinnon JA, He Y, et al Linking the National Health Interview Survey with the Florida Cancer Data System: A Pilot Study. J Registry Manag. 2016;43(1): 16–22. 27195994 PMC5682620

[16] Miller E, Miller D, Hudson D, He Y, Day H, Zevallos K, et al Linkage of 1986–2009 National Health Interview Survey with 1981–2010 Florida Cancer Data System. National Center for Health Statistics. Vital Health Stat. 2014;2(167). 25406513

[17] Fatalities among volunteer and career firefighters—United States, 1994–2004. MMWR Morb Mortal Wkly Rep. 2006;55(16): 453–5. 16645570

[18] Kahn SA, Leonard C, Siordia C. Firefighter Fatalities: Crude Mortality Rates and Risk Factors for Line of Duty Injury and Death. J Burn Care Res. 2019;40(2): 196–201. 10.1093/jbcr/iry037 30032307

[19] Petersen KU, Pedersen JE, Bonde JP, Ebbehoj NE, Hansen J. Mortality in a cohort of Danish firefighters; 1970–2014. Int Arch Occup Environ Health. 2018;91(6): 759–66. 10.1007/s00420-018-1323-6 29808435

